# An *in silico* pan-genomic probe for the molecular traits behind *Lactobacillus ruminis* gut autochthony

**DOI:** 10.1371/journal.pone.0175541

**Published:** 2017-04-17

**Authors:** Ravi Kant, Airi Palva, Ingemar von Ossowski

**Affiliations:** Department of Veterinary Biosciences, Faculty of Veterinary Medicine, University of Helsinki, Helsinki, Finland; Wilfrid Laurier University, CANADA

## Abstract

As an ecological niche, the mammalian intestine provides the ideal habitat for a variety of bacterial microorganisms. Purportedly, some commensal genera and species offer a beneficial mix of metabolic, protective, and structural processes that help sustain the natural digestive health of the host. Among these sort of gut inhabitants is the Gram-positive lactic acid bacterium *Lactobacillus ruminis*, a strict anaerobe with both pili and flagella on its cell surface, but also known for being autochthonous (indigenous) to the intestinal environment. Given that the molecular basis of gut autochthony for this species is largely unexplored and unknown, we undertook a study at the genome level to pinpoint some of the adaptive traits behind its colonization behavior. In our pan-genomic probe of *L*. *ruminis*, the genomes of nine different strains isolated from human, bovine, porcine, and equine host guts were compiled and compared for *in silico* analysis. For this, we conducted a geno-phenotypic assessment of protein-coding genes, with an emphasis on those products involved with cell-surface morphology and anaerobic fermentation and respiration. We also categorized and examined the core and accessory genes that define the *L*. *ruminis* species and its strains. Here, we made an attempt to identify those genes having ecologically relevant phenotypes that might support or bring about intestinal indigenousness.

## Introduction

In terms of ecological preference, the mammalian gastrointestinal (GI) tract is a hospitable biological niche for many different microorganisms, offering both mesophilic growing temperatures and a continual supply of nutrients and fluids [1]. Then again, the digestive system also has some challenging obstacles to circumvent, such as the high acidity of gastric juices, the emulsifying activity of bile, the recurring renewal of the mucosal epithelium, and the physical churning and peristalsis of the upper and lower intestines [2]. Accordingly, individual gut-dwelling bacteria have had to evolve a genome capacity that provides the phenotypic plasticity for the adaptive strategies considered necessary for surviving and enduring these environmental conditions. Accompanying this, the diverse assortment of bacterial genera and species residing in the intestine have come together to form a quasi-functioning communal unit (better known as a gut microbiota) that generates an intertwined network of symbiotically mutualistic relationships [3], which as an unintended outcome also provides ancillary benefits to the human or animal host [1, 4, 5]. Stemming from various adaptations and traits, this microbial consortium is mainly populated with so-deemed “intestinal friendly” commensals and occasional probiotics, although in some dire situations it can as well contain invading pathogens or opportunistic pathobionts [6]. Owing to the inherent properties of each bacterial type, gut colonization can be classified according to duration and longevity, and thus defined as either autochthonous (indigenous) or allochthonous (transient) [7, 8]. However, despite the GI tract being perceived as an open two-ended ecosystem, oxygen levels are variable and highly dependent on location, with graded concentrations running longitudinally and radially through the length of the intestine [9]. This subsequently delivers a range of microoxic-anoxic microenvironments for bacterial growth, which can be found scattered throughout various pocketed regions of the lumen and microvilli [9]. Predictably, some of the more prevalent bacteria in the early development of the gut microbiota are the facultative and strict anaerobes [10], with most becoming strongly entrenched and persistent intestinal inhabitants [3].

The Gram-positive genus *Lactobacillus* is grouped with the lactic acid bacteria (LAB) and includes upwards of 200 species (http://www.bacterio.net/lactobacillus.html). Noteworthy among these is *Lactobacillus ruminis* [11, 12], a strict anaerobe and one of the few lactobacilli to be described as “truly” autochthonous to the human and animal GI tract, as it persists during the entire lifetime of a host [13, 14]. Here, *L*. *ruminis* prevails over other types of gut-transient lactobacilli [15], most significantly in pigs [16]. Metabolically, *L*. *ruminis* is itself categorized with the obligate homolactic lactobacilli [17]. However, like many other gut bacteria, *L*. *ruminis* has adapted its metabolism to the nutrient conditions of the intestinal lumen, and through a capacity to utilize various carbohydrates [18, 19], it forages for energy as a prodigious saccharolytic fermenter. As cellular movement is uncharacteristic of most lactobacilli, with just a dozen or so species exhibiting a motility phenotype [20], it is notable that amongst these is *L*. *ruminis*. In particular are those strains obtained from bovine, porcine, and equine gut sources, where a variable number of lengthy flagella are used for cell propulsion, this manifested as either swimming in runs or swarming on solid surfaces [19, 21, 22]. As well, some human strains represent non-motile variants, but where flagellar movement seems to be reinvigorated to a tumbling-type once *L*. *ruminis* is isolated again after repassage through gut-environs [22]. Interestingly, *L*. *ruminis* was recently revealed as only the second *Lactobacillus* species whose cells jut out sortase-dependent pili [23]. As typically encountered with this type of surface piliation, these elongated protrusions (called LrpCBA) display an archetypal multi-subunit structure consisting of backbone, basal, and tip pilin-proteins [23]. Functionally, the LrpCBA pilus has a strong adhesiveness toward extracellular matrix (ECM) proteins (collagen and fibronectin) and intestinal epithelial cells [23], although unlike the SpaCBA [24–26] and SpaFED [27] pili from the *Lactobacillus rhamnosus* species, it lacks a substrate affinity for colonic mucus [23]. As a measure of eco-niche fitness, cell-surface morphologies like flagellation and piliation can emerge as rather advantageous to the *L*. *ruminis* species in promoting its permanent occupancy of the mammalian GI tract. For instance, these lengthy macromolecular appendages would provide the means by which *L*. *ruminis* cells can physically reach and colonize the concealed anoxic pockets of the highly folded epithelium in the upper intestine [23]. In addition, since some findings suggest that these particular flagellar and pilus structures arouse opposite effects of host immune-cell responsiveness [21–23], they might have a homeostatic counterbalancing role in the way *L*. *ruminis* is immuno-tolerated in the gut. Potentially, such evasion of the immune system could be partly causal in supporting an autochthonous and commensal lifestyle [23].

Presently, all known *L*. *ruminis* isolates are derived from the gut milieu, with no evidence to date suggesting this species dwells or can be sourced anywhere else, be it bodily or otherwise (such as food, vegetation, or soil) [19]. This contrasts with various other individual *Lactobacillus* species, which can be recovered from multiple host and environmental sources [28]. For those species in the mammalian gut, most tend to be allochthonous types that are eventually flushed out with the fecal stream [29–31]. Presumably, what causes the *L*. *ruminis* species to be a gut-indigenous bacterium is largely borne out by its adaptive ability to exploit and sustainably occupy a distinctly localized ecological niche in the host intestine. For this to occur, the *L*. *ruminis* genome would have needed to evolve and encode a number of genetic traits to suit the prevailing microenvironment. Here, in addition to overcoming certain physiological constraints for surviving within the GI tract (acidity, variable oxygen levels, and highly carbohydrous content), *L*. *ruminis* has acquired the necessary phenotypes for adhering to intestine-specific epithelial tissues, e.g., as provided by its LrpCBA piliation [23]. While some lactobacilli have a mucoadhesive nature that would favor cellular binding to the gut mucosal epithelium [32], the corresponding outer mucus layer is subject to regular removal and revitalization [33], thereby making any related bacterial occupation only temporary and short-term. By contrast, *L*. *ruminis* has taken another approach to gut habitation and eluded such a transitory fate by not only being an ineffective mucus binder, but having instead targeted its primary adhesion to ECM proteins [23], which represents the more hidden parts within the undulated epithelial lining where the routine shedding of cells is less a hindrance to colonization. Such tactical adaptation underscores the niche fitness advantage of cellular movement, as the flagellated motility of *L*. *ruminis* will help facilitate reaching this type of location for adhesion and subsequent growth, and by analogy, much in the same way that some pathogens can invade the gut [34].

At an *in silico* level, predictions from genomic data should be useful in pinning down some of the possible molecular mechanisms underlying *L*. *ruminis* gut autochthony. To date, just a few studies concerning the comparative genomics of *L*. *ruminis* have been published, but these are either broadly based [21] or involve a genetic emphasis on carbohydrate utilization and motility [18, 19]. Nonetheless, for our present study of *L*. *ruminis*, we instead opted to perform a pan-genomic probe into the natural adaptations that lie behind its colonization of the GI tract, and thus determine to what extent they involve genome evolvability and diversity. Here, the accompanying geno-phenotypic assessment takes a two-pronged approach that focuses on the cell-surface morphology and the anaerobic fermentative and respiratory processes, as each might contribute to the intestinal indigenous lifestyle of *L*. *ruminis*. To this end, the *L*. *ruminis* pan-genome was compiled from nine different genomes, three of which we had sequenced, and the rest retrieved from the public NCBI database. Further, these genomes originated from both ruminant and non-ruminant hosts. Ultimately, by using data from the pan-genome of *L*. *ruminis*, we categorized the essential core and dispensable accessory genes that define this bacterial species and its strains, and as well uncovered the commonality of some ecologically pertinent phenotypes that potentially contribute to a gut-autochthonous character.

## Materials and methods

### Genome sequencing, assembly, and annotation

*L*. *ruminis* bovine strains PEL65 (also called DSM 20403 or ATCC 27780) and PEL66 (also called DSM 20404 or ATCC 27781) were purchased from the DSMZ culture collection (Braunschweig, Germany). Porcine strain GRL1172 was obtained from our in-house culture collection (Department of Veterinary Biosciences, University of Helsinki). High-throughput next-generation sequencing of the genomes from the PEL65, PEL66, and GRL1172 strains was carried out at the Institute of Biotechnology (University of Helsinki, Finland) using a standard approach described previously [35]. Briefly, *L*. *ruminis* cells were grown and pelleted [23], and their genomic DNA extracted with commercial kits. Shearing of genomic DNA into random fragments (3 μg per 100 μl) was performed with a Covaris S2 acoustic sonicator (Covaris Inc., USA). In a 50-μl volume, DNA fragments were then fractionated into uniform 1.2-kb sizes using magnetic carboxyl beads, as done previously [36], followed by end polishing and 454 Y-adapter ligation using the method of the manufacturer (Roche/454 Life Sciences, USA). Emulsion PCR was employed for amplifying the genomic libraries, which were sequenced using the Roche Genome Sequencer FLX+ system. Following this, sequence reads were assembled *de novo* into contigs with GS Assembler software (Roche/454 Life Sciences, USA). Draft assemblies of the genome sequences were submitted to GenBank with accession numbers MRYP00000000 (PEL65), MRYQ00000000 (PEL66), and MRYO00000000 (GRL1172). For generating the pan-genome assembly, an additional six *L*. *ruminis* genomes (strains DPC 6830, DPC 6832, ATCC 25644, SPM0211, S23, and ATCC 27782) were retrieved from the NCBI RefSeq database (as of July 2016). For those genomes in the database with more than one sequenced version, a representative one was randomly chosen, this having been done to avoid any potential skewing of the data output. Draft genome assemblies of the PEL65, PEL66, and GRL1172 strains were annotated individually by using the RAST (Rapid Annotation using Subsystem Technology) automatic annotation pipeline [37], with some minor manual curation being done afterward. Pre-existing gene annotations were downloaded and used for the six genome sequences obtained from the NCBI RefSeq database.

### Orthologous gene identification

Orthologous genes were identified as an all-to-all gene comparison of every *L*. *ruminis* genome using a BlastP algorithm [38] with a BLOSUM62 default-scoring matrix and an *E*-value cut-off set to 10^−4^. Normalizing of the raw BLAST hit scores against the maximum possible score (defined here as the self-hit score for each gene) was performed. As the BLAST score ratio values (SRV) can then be in a 0–100 range, this will better reflect the “hit quality” as opposed to the raw BLAST bit-scores [39]. Correspondingly, orthologs represent two loci if a mutual pair-wise reciprocal best BLAST hit (RBBH) is obtained, and if the SRV is ≥ 35 for each of the hits.

### Determination of the pan-, core, and accessory genomes

The pan-genome was estimated by additive comparisons of the various *L*. *ruminis* genomes, in which collections of non-orthologous loci are consecutively added to the set of genes from a reference genome (GRL1172) randomly selected for pan-genome assembly. Estimation of the core genome involved a succession of reductive comparisons between the various *L*. *ruminis* genomes, with these representing the orthologous loci in the nine genomes. Functional classifications were assigned to the putative core proteins by performing a BLAST search against sequences in the COG (clusters of orthologous groups) database (https://www.ncbi.nlm.nih.gov/COG/) [40]. The accessory genome, which instead represents the non-orthologous loci in the pan-genome, consists of those genes shared by a minimum of two *L*. *ruminis* genomes, but not all them. Unique (or strain-specific) genes are a subset of accessory genes and appear in only one of the genomes. Comparative analyses that generated orthology-related data were done with EDGAR software [41]. Development plots of the pan-genome and core genome for the nine *L*. *ruminis* strains were computed using R statistical programming language and Heap’s Law. Estimated values for the κ, γ, and α parameters were extracted from a graphical curve optimized for a nonlinear least square fit of the genome data [42, 43]. In-house scripts were used when deemed necessary.

### Comparative phylogenomic analysis

Genomic relatedness between or among the nine *L*. *ruminis* strains was assessed for evolutionary relationships using an earlier described phylogenetic tree reconstruction method [35, 44]. Here, a core genome phylogeny was inferred using the approach taken by Zbodnov and Bork (2007) [45], wherein orthologous genes in different genomes are identified according to their protein homology predictions. For this, MUSCLE [46] was used to construct multiple genome alignments of the in-common core genes, and subsequent to the concatenation of the alignment blocks, GBLOCKS [47] was used for deleting away the sequence gaps and misaligned sections. Unrooted neighbor-joining phylogenetic tree building of the multiple genome alignments was done using PHYLIP [48].

### *In silico* protein prediction

*In silico* estimates for the proteinaceous architecture of the *L*. *ruminis* cell surface were done using openly available protein prediction tools, either with an online server or when downloaded. In some instances, in-house scripts were used to complement the various algorithmic predictions. For all protein prediction programs, loci from the entire pan-genome were analyzed, but with the exclusion of predicted products whose primary structure is ≤ 45 amino acids. When discrepancies arose, manual inspection of the prediction data output was performed. Depending on which of the predictive analyses was undertaken, some data output was sorted into either the core or accessory genomes. For identifying N-terminal (classical) secreted proteins, predictions for the occurrence of Sec-dependent and twin-arginine translocation (Tat) signal peptides were made using SignalP 4.1 (http://www.cbs.dtu.dk/services/SignalP/) [49] and TatP 1.0 (http://www.cbs.dtu.dk/services/TatP/) [50], respectively. For the SignalP predictions, default settings for Gram-positive bacteria and a cut-off score of ≥ 0.45 were used. Non-classical secreted proteins were identified by using the SecretomeP 2.0 prediction program (http://www.cbs.dtu.dk/services/SecretomeP/) [51]. For this, those proteins having a recommended SecP score of ≥ 0.5 were judged to be potentially secreted. TMHMM 2.0 (http://www.cbs.dtu.dk/services/TMHMM/) [52] was used to identify which protein loci are encoding transmembrane helical regions and PRED-LIPO (http://biophysics.biol.uoa.gr/PRED-LIPO/) [53] was used to predict any putative lipoproteins. Detection of possible sortase-dependent proteins (SDPs) was done with the CW-PRED method (http://bioinformatics.biol.uoa.gr/CW-PRED/) [54], which predicts the presence of an intact C-terminal LPXTG (or LPXTG-like) cell wall-anchoring domain.

## Results and discussion

### General attributes of the *L*. *ruminis* genomes

*L*. *ruminis* genome sequences of nine gut isolates were used and analyzed in this study. Here, genomes were sourced from bovine (*n* = 3), human (*n* = 3), porcine (*n* = 2), and equine (*n* = 1) hosts, these representing both the ruminant and non-ruminant digestive systems. Among the genomic sequences, six genomes (DPC 6830, DPC 6832, ATCC 25644, SPM0211, S23, and ATCC 27782) were obtained from the NCBI RefSeq database, whereas we supplied the remaining three genomes (GRL1172, PEL65, and PEL66) via high-throughput sequencing. For this latter set of genomes, the assembled draft sequences were initially annotated using an automated pipeline for gene identification, and then afterward improved by extra manual curation. Plasmid DNA sequence was excluded from this annotation process. A list of the annotated genes predicted for the three newly sequenced genomes is given as supporting information (S1 Table), with each genomic sequence having been deposited into GenBank (see Materials and Methods for accession numbers). Summarized in Table 1 are the general attributes of the nine genomes used for compiling the *L*. *ruminis* pan-genome. Despite the fact the majority of these genomes are draft assemblies, with only the one from the bovine ATCC 27782 strain being sequenced to completion; they still represent suitably good quality sequence data for conducting genomic comparisons. Here though, the average coverage of the genome sequencing ranges widely from 16-fold (GRL1172) to 1500-fold (SPM0211), but this can be largely attributed to the use of various sequencing platforms. Moreover, the number of contigs in the assembled genomes is spread out between one and 422 (as in ATCC 27782 and S23, respectively). *L*. *ruminis* genome sizes range between 1.91 (S23) and 2.17 (SPM0211) Mbps, and then averaging out at 2.04 Mbps. While the sizes of the genomes from the porcine and bovine hosts are each closely similar, the human-derived genomes show greater size variability. Even so, the GC base pair content for the nine genome sequences varies only slightly and is between 43.1 and 43.7%. Further, whereas the number of identified open reading frames (ORFs) in the nine genomes ranges from 1845 to 2194, those predicted to encode proteins number between 1686 and 2105. This equates to an averaged overall difference of 17.3 and 22.1%, respectively. As these data suggest a reasonable similarity in the genomes of the strains, *L*. *ruminis* can be regarded as a genetically homogeneous species.

**Table 1:** Overview of the *L*. *ruminis* genome sequences in this study.

Strain	GRL1172	DPC 6830	DPC 6832	ATCC 25644	SPM0211	S23	ATCC 27782	PEL65	PEL66
**Gut Source**	Porcine	Porcine	Equine	Human	Human	Human	Bovine	Bovine	Bovine
**BioProject**	PRJNA357347	PRJNA239217	PRJNA219505	PRJNA31499	PRJNA67675	PRJNA219503	PRJNA70721	PRJNA357347	PRJNA357347
**Platform**	Roche 454 GS FLX	Illumina HiSeq 2000	Illumina HiSeq 2000	Roche 454 GS FLX	Illumina GA IIX	Illumina HiSeq 2000	Roche 454 GS FLX ^a^	Roche 454 GS FLX	Roche 454 GS FLX
**Status**	Draft	Draft	Draft	Draft	Draft	Draft	Complete	Draft	Draft
**Coverage**	16x	329x	1382x	39.72x	1500x	658x	28x	18x	20x
**Contigs**	83	89	128	58	12	422	1	65	54
**Size** ^**b**^ **(Mbps)**	2.02	2.04	1.94	2.11	2.17	1.91	2.07	2.06	2.02
**G+C (%)**	43.3	43.3	43.1	43.7	43.7	43.7	43.5	43.4	43.4
**ORFs** ^**c**^	2165	1975	1897	2072	2127	1845	2035	2194	2170
**Proteins** ^**d**^	2105	1825	1755	1968	2013	1686	1836	2104	2086
**Reference**	This study	[19]	[19]	Unpublished	[55]	[19]	[21]	This study	This study

^a^ Illumina HiSeq 2000 sequencing was also done for 22.5 Mb data and gave 217-fold coverage.

^b^ Average genome size is 2.04 Mbps.

^c^ Average number of ORFs is 2053.

^d^ Average number of proteins is 1930.

### Reconstructed phylogeny of *L*. *ruminis* genomes

**Fig 1:**
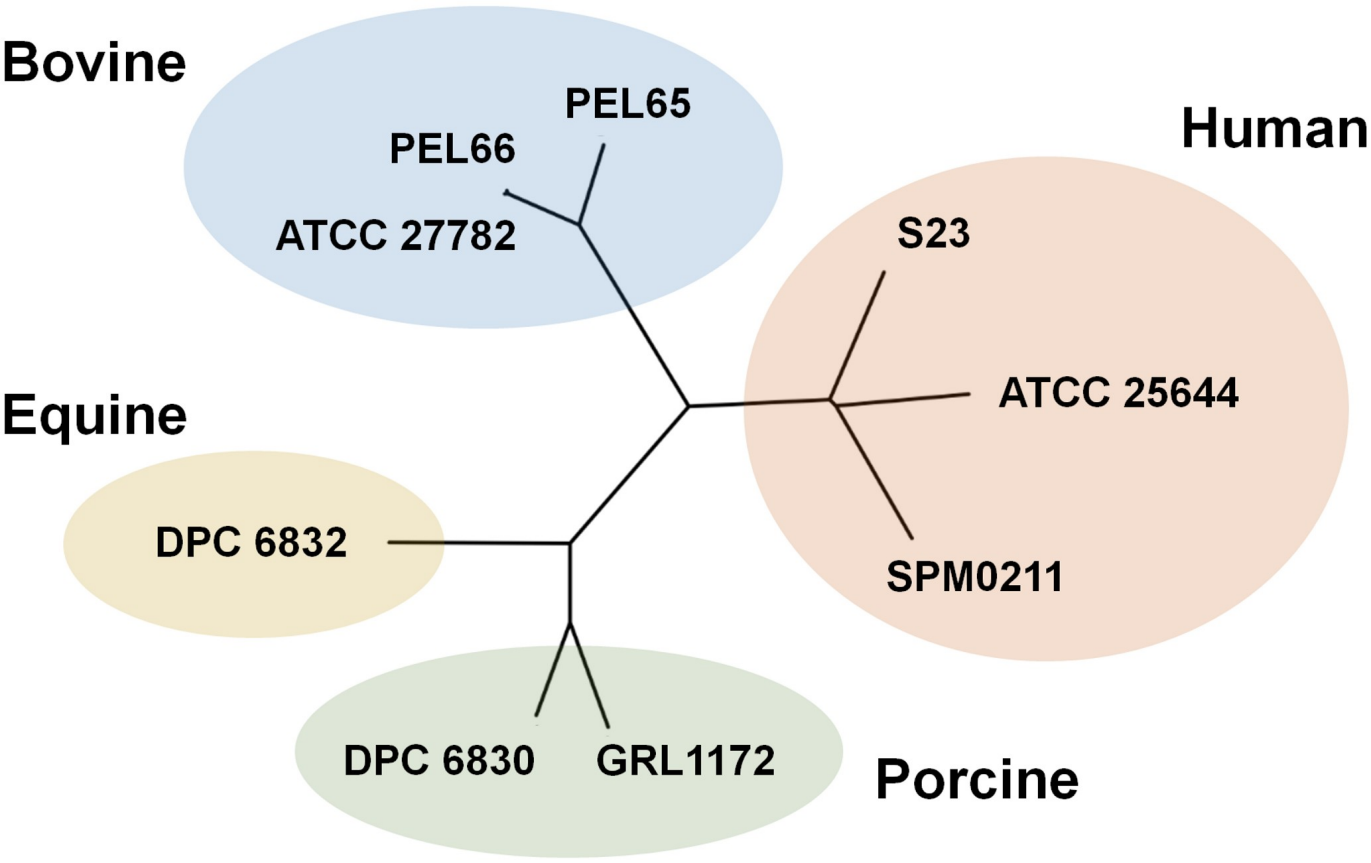
Phylogenetic tree analysis of the *L*. *ruminis* genomes. Unrooted phylogenies of the *L*. *ruminis* genomes were based on the multiple sequence alignment of core proteins and constructed with the neighbor-joining tree-building algorithm (see Materials and Methods for details). Names of the *L*. *ruminis* strains from which each genome is derived are shown. Four distinct phyletic clades were identified based on host-gut source (human, bovine, porcine, or equine) and are indicated accordingly.

Because the natural habitat of the *L*. *ruminis* species seems restricted to the gut milieu, we decided to conduct a phylogenetic reconstruction to gauge whether any genomic relatedness is reflected in a particular type of host source. For this, a phylogenomic tree of the nine *L*. *ruminis* strains was built using a multiple alignment of the 1234 loci (proteins) from the core genome. As shown in Fig 1, the various *L*. *ruminis* genomes sort out into distinct clades that are based on the host-derived origin of each isolate. Here, it was surprising to see a closer phylogenetic relationship between the genomes of the human and bovine *L*. *ruminis* strains, as this would imply a recent and common ancestry, this despite the host-gut origins being divergent. Specifically, humans utilize a monogastric digestive system, and one that is more similar to those found in pigs and horses [2]. By contrast, ruminant herbivores like cattle have a multi-chambered stomach, where nearly total digestion of grazed plant cellulose occurs in the reticulorumen pouch [2, 56]. From an ecological perspective, these two compartmentalized gastric niche locales would presumably offer different evolutionary challenges to the gut-dwelling *L*. *ruminis* species, and at a genome level likely demand that strains have a characteristic set of geno-phenotypes for adapting to such environments. However, based on Fig 1, it is rather unexpected that the phylogenomic lineage of the human *L*. *ruminis* strains would not be nearer to that of the porcine- and equine-derived isolates. Thus, even though *L*. *ruminis* phylogenomics support the various isolates as being host-source specific, there is no apparent commonality amongst the gene content of the core genome that causes a clear lineage distinction between the ruminant-derived strains and those isolated from non-ruminating monogastric hosts. For the latter, it seems that the evolutionary potential of the *L*. *ruminis* genome is not selectively shaped or constrained by a distinguishably dominant factor in these two differing gut habitats.

### Assembling the *L*. *ruminis* pan-genome

**Fig 2:**
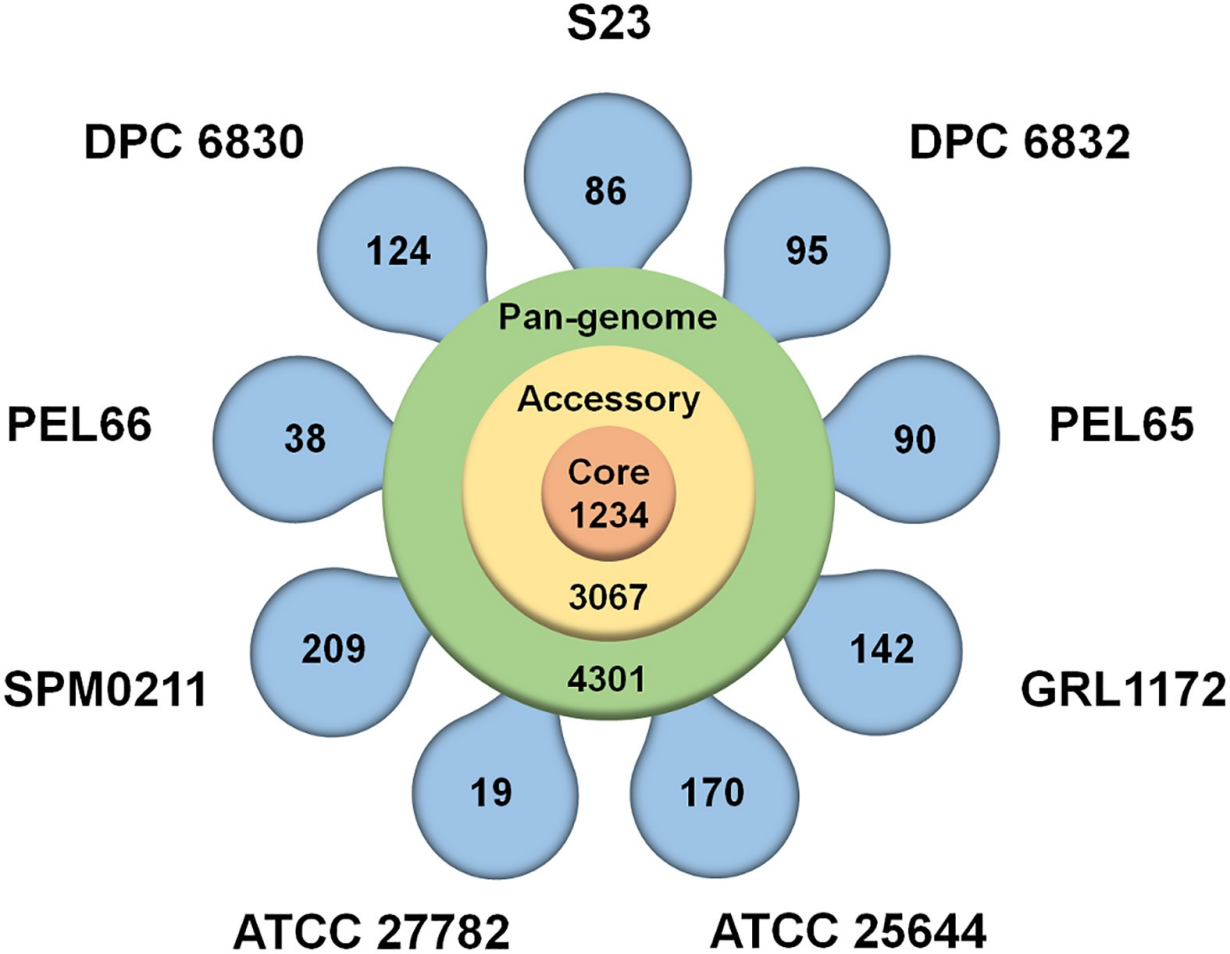
Pan-genome of the nine *L*. *ruminis* strains. A flower-plot depicting the gene content in the pan- (4301), core (1234), and accessory (3067) genomes of *L*. *ruminis* is shown. The number of strain-specific genes per genome is shown in the flower petals. Names for each *L*. *ruminis* strain are indicated. Annotated core and accessory genes are provided in the S1 Table.

**Fig 3:**
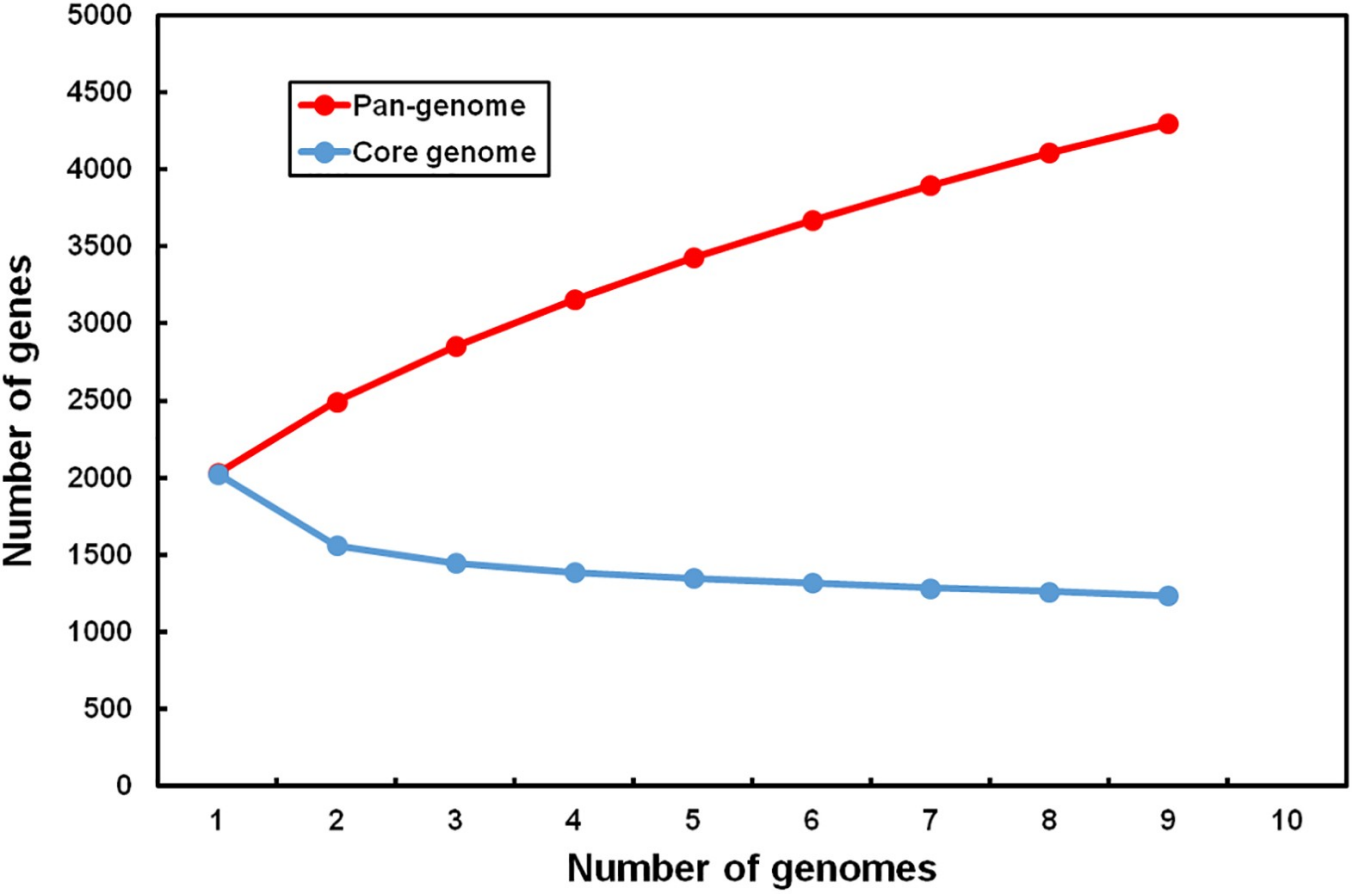
Size prediction for the *L*. *ruminis* pan- and core genomes. New gene numbers in the pan- and core genomes are plotted against the number of added *L*. *ruminis* genomes. Each fitted curve was generated using the median values for the number of new genes.

For our study, a pan-genome was created using the genomes of the nine *L*. *ruminis* strains described in Table 1. Here, the total genetic repertoire was deduced from computed BLAST score ratio values (SRVs), these having been determined within the EDGAR software framework. The *L*. *ruminis* pan-genome is estimated to contain 4301 protein-related genes (Fig 2 and S1 Table), and thus the genetic pool maintained by this species is slightly more than double (2.2-fold) the average number of 1930 predicted protein loci for the nine genomes. When a Heap’s Law calculation (for a description, see [42, 43, 57, 58]) is extracted from a plot of the pan-genome gene number versus the number of *L*. *ruminis* genomes being considered (Fig 3), the α-value that was obtained is only 0.63. As α is < 1, this convincingly implies that the pan-genome is very much open [42, 43, 57, 58] and any related accessible gene pool is far from being completely characterized for the *L*. *ruminis* species. As a mathematical extrapolation, the pan-genome curve shows no signs of leveling out and appears to project unlimited gene content (Fig 3). On the other hand, as the curve trajectory of the development plot for the core genome is sharply lowered to a plateau, it seems evident that there is a progression toward a stable number of core genes (Fig 3). This underscores the importance of the gene content in the accessory genome for the environmental adaptability of the *L*. *ruminis* species (see forthcoming sections for more details).

### Core genome of *L*. *ruminis*

**Fig 4:**
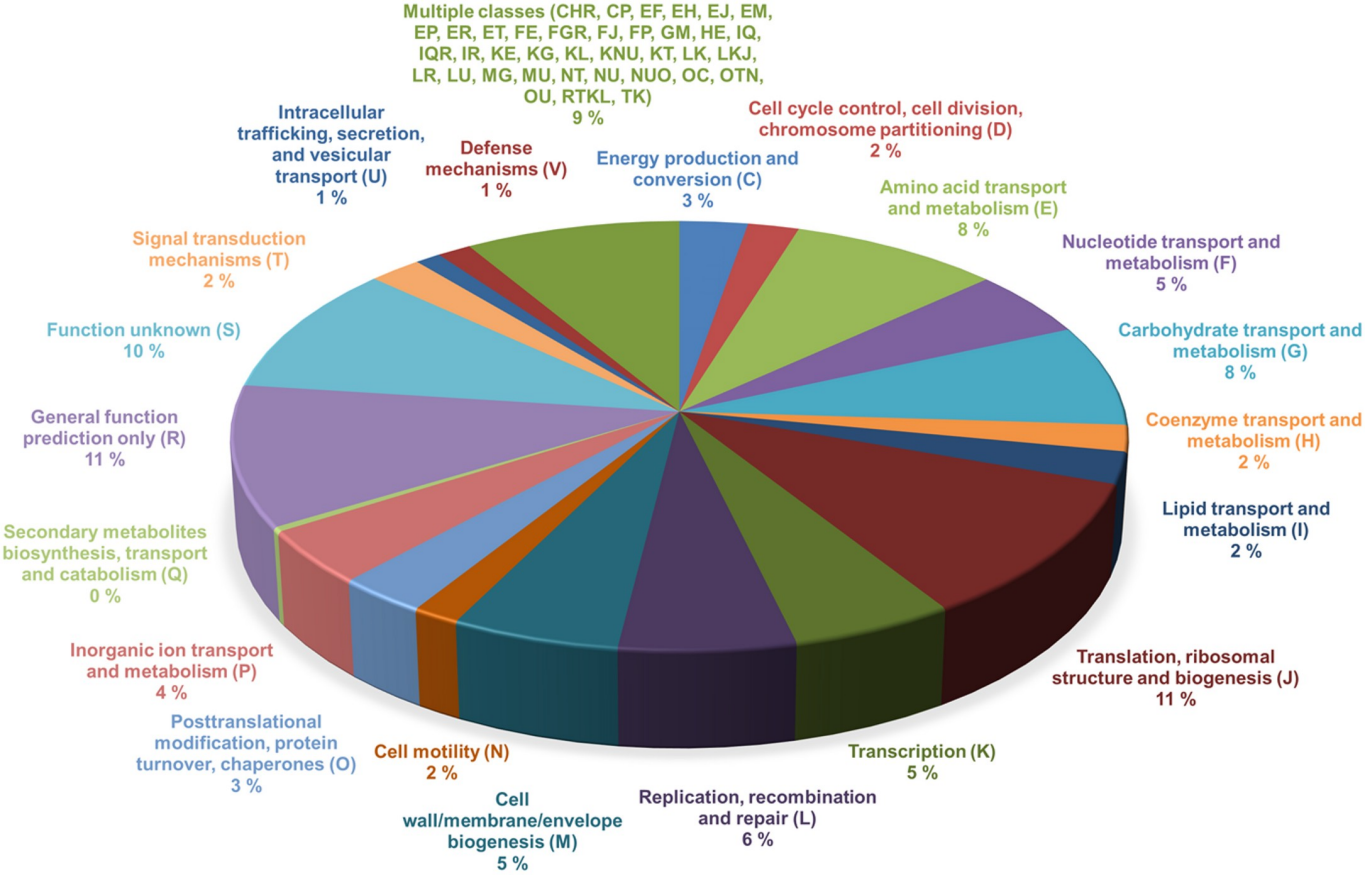
Functional classifications of the *L*. *ruminis* core genome. COG (clusters of orthologous groups) functional classifications were assigned to the putative proteins of the *L*. *ruminis* core genome as described in Materials and Methods. Various core proteins were categorized according to 21 COG functional assignments as a percent of the total number of predicted proteins in the core gene pool. Results are shown as a pie chart, with the names and abbreviations for the COG categories and the corresponding percentages (%) indicated.

The various loci of the pan-genome that are deemed crucial and necessary for the self-sustainability of a bacterial cell are what encompass the core genome of a particular species. In general, among the predicted ORFs for the core genes are those assigned to basic housekeeping functions and regulatory mechanisms. These broadly cover the different catabolic and metabolic pathways for the cellular production, transport, utilization, and degradation of nucleic acids, ribosomes, and proteins, but as well, other compounds or molecules such as carbohydrates, amino acids, and lipids [42, 43, 57, 58]. Predictably, the core genome of *L*. *ruminis* (S1 Table) mirrors the established consensus, with its collection of loci falling into a similar set of categories described above and as are now itemized in Fig 4. Here, the COG (clusters of orthologous groups) distribution of the core genes illustrates how the corresponding putative proteins are assigned and classified according to their possible function. For these COG classification estimates, some of the functional categories that emerge with the higher percentage of core genes are predictable and include those dealing with the transport and metabolism of carbohydrates (8%), amino acids (8%), and nucleotides (5%), the biogenesis of the cell envelope (5%), and as well various translational (11%), transcriptional (5%), and DNA replication-related (6%) processes. However, there is also a large number of core loci that are categorized with multiple classifications (9%), general function predictions (11%), and unknown functions (10%). Of particular interest, given that *L*. *ruminis* strains have a proclivity for flagellated cellular movement, it is both expected and logical that a certain proportion (2%) of the core genome would be devoted to a functional classification for cell motility (see latter section for more details). As for its overall estimated size, the *L*. *ruminis* core genome converges to 1234 protein-encoding loci (Fig 2 and S1 Table), which equates to just 28.7% of the pan-genome size (4301) and, parenthetically, to about two-thirds the average number of protein genes (1930) for the nine genomes of the pan-genome (Table 1). This relatively low number of core loci implies that this species retains a wide-ranging genome structure, which by inference, would mean that the accompanying accessory or dispensable genome is somewhat large sized.

### Accessory genome of *L*. *ruminis*

In addition to having a conserved genetic core, the *L*. *ruminis* pan-genome includes a supplementary pool of loci, with these encompassing the so-called accessory genome and reflecting the diversity of this lactobacillar species. While the accessory genes correspond to those that are partly shared (at least two genomes but not all) or unique (strain-specific), and are often dispensable, some tend to help customize bacterial strains for adaptation and survival in certain environmental settings and therefore might be causally linked to a particular ecological lifestyle or habitat [42, 43, 57, 58]. Based on our estimates, the *L*. *ruminis* accessory genome is itself rather amply sized and consists of 3067 protein genes (Fig 2 and S1 Table), which, by calculation, is nearly 2.5-fold greater than that of the core genome and close to covering 70% of the pan-genome gene pool. By comparison, in our previous pan-genome study of *L*. *rhamnosus* [35], a species that has adapted to a broad range of ecological habitats, the corresponding accessory genome was found to represent ~57% of the genes in the pan-genome. Nonetheless, the relatively high accessory gene content for *L*. *ruminis* is reflective of the genetic diversity that would be needed by this species to subsist in a specific yet physically dynamic locale like the mammalian gut.

Among the loci of the accessory genome there exist those that are unique and specific to an individual *L*. *ruminis* strain. For these genes, their numbers per genome are provided in Fig 2, the most being found with the human SPM0211 strain (209 loci) and the least from two bovine isolates (19 and 38 loci in ATCC 27782 and PEL66, respectively). The overall number of strain-specific genes adds up to 973, which proportionally accounts for 31.7% of the accessory genome. By far the majority of these unique loci have annotations for hypothetical proteins (S1 Table), and as most variation then lies with unknown and uncharacterized functionalities, it is difficult to correlate any type of adaptive role or benefit for the nine different *L*. *ruminis* strains. Nonetheless, for a few of the strains some of their unique loci are annotated with a variety of predicted functions in transport and metabolism, but recognizably as well, for those involved with the lateral transfer of genes, i.e., phage-related proteins, transposases, and mobile elements (S1 Table).

### Proteinaceous content within the cell-surface architecture of *L*. *ruminis*

Universally, the outer surface morphology of Gram-positive bacteria encompasses a complex mix of different proteinaceous and carbohydrous structures (for detailed reviews, see [59–62]). Typically, when bacteria confront the peripheral environment, it is presumed that their surface-exposed features will have certain protective or aggressive functions, and thus involve a variety of cellular binding, recognition, and signaling actions. In the subsections that follow, the pan-genome was used to reveal the cell-surface architecture of *L*. *ruminis*, with an overall emphasis on the predicted proteinaceous character. This specifically includes an examination of the loci encoding classical and non-classical secretory proteins, transmembrane proteins, lipoproteins, LPXTG-anchored surface proteins, and the flagellar and chemotaxis proteins. Here, an effort was made to assess whether any variations in these cell surface-related proteins can define the ecological niche preference of each individual *L*. *ruminis* strain.

#### Classical and non-classical secretory proteins

Various proteins that populate the outer surface of Gram-positive bacteria entail some sort of signaling peptide to help facilitate their translocation across the cell envelope barrier. For instance, the route of N-terminal signaled “classical” protein secretion can occur through either the Sec-dependent or twin-arginine translocation (Tat) pathways. Then again, some secreted proteins use “non-classical” pathways and do so without any obvious signal peptides. For our *in silico* predictions, the SignalP [49], TatP [50], and SecretomeP [51] programs were used to discriminate the number and type of exported proteins utilizing the classical pathways from those that are secreted by non-classical means. Predicted primary structures of loci from all nine *L*. *ruminis* genomes were individually processed per genome and the output analyzed afterward. Data from the SignalP and TatP predictions were also sorted into either the core or accessory genomes.

According to the SignalP results (S2 Table), it appears that classically secreted proteins requiring a Sec-signal peptide account for a maximum of only 75 putative loci (ATCC 25644). Among these protein genes, an estimated 33–48 belong to the core genome (equivalent to 2.7 to 3.9%, respectively). Of note, we attribute the range in pooled core genome number as a general inconsistency with *in silico* prediction programs. For example, with SignalP, this can also partly originate from some of the “secreted” protein loci unexpectedly lacking a deduced primary structure for an intact N-terminal region, which causes them to be missed and not predicted. For the Sec-signaled proteins that are encoded by non-core loci, these number from six to 29 genes and average out to less than one percent of the accessory genome (S2 Table). Prediction estimates using the TatP method indicate that there is a minimum of 15 (DPC 6832) and a maximum of 38 loci (SPM0211) with encoded proteins that would have the sequence potential for a Tat signal peptide (S3 Table). Of these, the designated core genes are between 11 (ATCC 27782 and DPC 6830) and 14 (PEL65 and S23), whereas those loci that make up the accessory genome range from three to 26 in number (DPC 6832 and SPM0211, respectively). By comparison, the SecretomeP prediction data gives an approximation of loci for non-classically secreted proteins that is at least ten-fold greater in number. In total, there are between 526 and 841 putative loci whose encoded products presumably use one or other unconventional routes for exporting proteins that do not have a typical N-terminal signaling peptide (S4 Table). While these numbers of protein loci might appear high, we consider them to be acceptable given that the recommended SecP score (≥ 0.5) for indicating secretion was used in our determinations [51].

Our survey of the corresponding annotations for the putative classical and non-classical secretory proteins indicates that for both these types many of them have some link to a recognizable extracellular function. In the context of cellular adhesion, one of the non-classically secreted proteins is worthy of mention (S4 Table). Here, it might be envisioned that active expression of the core gene annotated as fibronectin-binding protein (Fbp) (GRL1172_498, HMPREF0542_10570, LRC_RS05075, LRN_0851, PEL65_1842, PEL66_466, P869_04425, LRU_00261, and LRP_1613) might contribute to the gut autochthony of the various *L*. *ruminis* strains. Fibronectin is an ECM glycoprotein found near the surface of epithelial cells and can serve as a target or attachment site for many types of bacteria, including a variety of *Lactobacillus* species [63, 64]. For the binding of *L*. *ruminis* cells to gut-localized fibronectin, this might be mediated through the Fbp, along with the LrpCBA pilus, which has been shown to have similar substrate specificity [23]. As with other lactobacillar counterparts [35, 65], *L*. *ruminis* Fbp is atypical from those in pathogens and is seen to lack any familiar signaling domains at its C- or N-termini, thus explaining why this protein was detected with the SecretomeP method.

#### Transmembrane proteins

In Gram-positive bacteria, many of the proteins that are being exported through the cell envelope also possess hydrophobic regions that facilitate membrane insertion, but as well, where other regions extend beyond the cell wall to the outer surface. These transmembrane proteins (as they are classified) contain one or more membrane-spanning helical regions whose hydrophobicity allows them to be embedded in the cytoplasmic membrane. Functionally, Gram-positive transmembrane proteins have varied purposes, these ranging from the metabolism of macromolecules (polysaccharides, nucleic acids, proteins, and lipids) to different cellular signaling processes and gene regulation activities [60]. By using the TMHMM algorithm [52], which predicts the presence of transmembrane helices (TMHs), we were able to gauge which of the *L*. *ruminis* genes from the pan-genome are representing putative transmembrane proteins (S5 Table). In total, we found there were between 308–480 hits (DPC 6832 and ATCC 25644, respectively) for protein loci that are predicted to encode transmembrane helical regions, but where 222–341 of these predictions are for proteins containing two or more TMHs. While not unexpected, functional annotations for these transmembrane proteins were in line with other Gram-positive bacteria. Interestingly, the maximum number of predicted TMHs per encoded protein was found to be 14, and of these integral membrane proteins they accounted for five to eight loci, most of which were annotated as various members of the transporter protein family.

Among the transmembrane protein hits, we identified an ORF in two human strains (HMPREF0542_11925 from ATCC 25644 and P869_03355 from S23) that encodes for a possible fucose permease (FucP) [66]. This transporter protein is responsible for the cellular uptake of fucose, a hexose sugar commonly found as a glycan component of the mucin proteins forming the mucus layer of the gut epithelium, and which for certain bacteria can serve as an energy substrate instead of glucose should nutrients become limiting [67]. Noticeably, none of the two genomes contains the gene encoding for an α-fucosidase, the enzyme that would hydrolyze the release of fucose from the mucus glycan chain as found with *Bifidobacterium bifidum* [68]. This can explain the findings of another study where fucosidase activity was not detected in the ATCC 25644 and S23 strains [19]. Even so, fucose could instead be available as scavenged remnants in the gut, and subsequently absorbed into the cells via the putative fucose permease transporter. Thus, with the possibility of having the transport machinery that enables cells to feed on spent intestinal mucus, this can be regarded as a habitat-specific fitness advantage for these two human strains of *L*. *ruminis*. Interestingly, despite the fact that mucus-lined intestines are a universal feature of mammals, the fucose permease-encoding gene is not shared by the genomes of the other host-derived *L*. *ruminis* strains, and thus it belongs to the accessory genome.

#### Lipoproteins

Lipoproteins in Gram-positive bacteria represent a functionally diverse group of membrane proteins (transporters, enzymes, adhesins, and receptors) that protrude outwardly from their covalent anchoring at the interface region between the cytoplasmic membrane and the peptidoglycan layer of the outer cell wall [60]. Bioinformatically, Gram-positive lipoproteins can be readily recognized based on their so-called lipobox, a pattern of residues ([LVI]-[AST]-[GA]-C) in the C-region of its N-terminal secretion signal sequence that serves as the cleavage recognition site for signal peptidase II [60]. In our pan-genomic survey of *L*. *ruminis*, loci encoding putative lipoproteins were identified for the presence of a lipobox using the PRED-LIPO prediction program [53], wherein a total of between 16 and 30 genes had been uncovered (S6 Table). After sorting into the core and accessory genomes, 12–19 core and four to 13 non-core genes were found to exist. Aside from a few hypothetical protein annotations, the majority of the predicted lipoprotein genes are annotated with functions as ABC transporter proteins (or otherwise related), these including a variety of potential substrate binding abilities. Given their essential role in the exchange of metabolic products, such putative lipoproteins can be regarded as a necessary feature of the various *L*. *ruminis* strains and their evolved capacity to utilize the nutrient richness of the gut environment.

#### LPXTG-anchored surface proteins

Often exposed on the cell surface of many different Gram-positive genera and species are various types of proteins whose covalent cell wall anchoring relies on the action of transpeptidyl enzymes called sortases. Such sortase-dependent proteins (SDPs) require the presence of a C-terminal signaling region that includes a five-residue recognition and cleavage motif (LPXTG), which is itself followed by a stretch of aliphatic amino acids and a tail end of positive-charged residues (for detailed reviews, see [60, 62, 64]). To identify any potential loci for SDPs within the nine *L*. *ruminis* genomes, we made use of the CW-PRED algorithm, a Hidden Markov Model (HMM)-based tool that predicts the occurrence of LPXTG (or LPXTG-like) domains [54]. The output of these predictions revealed that per genome there is a minimum of three and maximum of seven hits for SDP loci. For these, each of the corresponding primary structures was visually inspected for an intact LPXTG domain region, whereupon one hit was judged an artifact and subsequently rejected. In total, nine types of SDPs were found, of which three loci are part of the core genome, with the six others being associated with the accessory genome (S7 Table). Surprisingly, although it is common for SDPs to also have a Sec-signal peptide at the N-terminus, not all of these nine protein loci were recognized within our SignalP prediction data (S2 Table). On the other hand, largely due to the predicted presence of an intact LPXTG domain, each of these genes was identified by using the TMHMM method (S5 Table).

Amongst the designated core genes (S7 Table), we had identified those encoding for the backbone (LrpA) and tip (LrpC) pilin subunits of the LrpCBA pilus [23]. As our earlier work revealed the LrpCBA pilus is a macromolecular structure and assembled from three different protein subunits [23], it was unexpected that the gene for the basal LrpB pilin would not be found in the core genome, particularly since the three LrpCBA pilin subunits are encoded by loci that are clustered and expressed together as a fimbrial operon (called *lrpCBA*) [23]. Of the nine *L*. *ruminis* genomes encompassing the core genome, the genome from the ATCC 27782 strain was identified as being the source of this discrepancy. After manually checking the NCBI database where the ATCC 27782 genome sequence is deposited, we found the corresponding LrpB locus tag (LRC_RS00315) had its ORF hidden as a pseudogene. Previously, under an old locus tag (LRC_00610), amino acid sequence for this ORF was attainable and upon inspection we found it represented a truncated version that was missing about 40 residues at the N-terminus, including those for a secretion signal peptide. Further, we noticed that this shortened LrpB primary structure results from an insertion of two adenines along the *lrpB* coding sequence, thus producing a reading-frameshift change. Relatedly, it is worth mentioning we had previously observed that for the DPC 6832-sourced LrpB pilin, there is a shift in the reading-frame of its deduced primary structure, which we found is due to a missing cytosine in a serine codon [23]. Consequently, during the annotation process the LrpB pilin was detected as two ORFs and had been assigned separate locus tags (LRN_0080 and LRN_0081). Despite the fact that LRN_0081 was confirmed as having the entire LPXTG domain region, it was the LRN_0080 ORF that became aligned with the other-sourced LrpB loci in the *L*. *ruminis* pan-genome, which for us led to another inconsistency during our interpretation of these results. However, for both types of detected nucleotide alterations, it is unclear if they arose from a genuine indel mutation or as an unintentional mistake in DNA sequencing. In all likelihood, there is the possibility that the *lrpB* gene would instead be better placed within the core genome of *L*. *ruminis*.

As for the third core gene whose ORF was predicted as having a LPXTG domain, it had been given various annotations (S7 Table), but none offered any sort of functional description other than being a cell wall-anchored protein. Since the amino acid identity between the various-sourced versions of this LPXTG surface protein is quite high (92.7–100%), we examined whether any homology occurs with recognized binding domains. Here, no hits were obtained from a Pfam search (at http://pfam.xfam.org; [69]) when using the HMPREF0542_11626 sequence of ATCC 25644. However, when performing a BlastP search of the NCBI database with the same primary structure, it was revealed that some similarity to the MucBP (Mucin-Binding Protein; pfam06458) domain exists, suggesting this cell wall-anchored protein might adhere to mucosal substrates. This possibility comes as somewhat unexpected, as previously we found that *L*. *ruminis* cells show little binding affinity for mucus [23].

Regarding the LPXTG-domained protein loci found within the accessory genome (S7 Table), three of them appear to be strain-specific (LRP_348 in porcine DPC 6830, and P869_01660 and P869_04430 in human S23). As far as any Pfam-predicted functions, no noteworthy matches were obtained from a database search, which is expected considering that all three genes are annotated as hypothetical protein. Also of note, two additional accessory genes are each common to two different strains. However, whereas the LRP_1521 (from DPC 6830) and PEL65_242 (from PEL65) ORFs are assigned with a starch-debranching pullulanase function, the LRU_00897 and P869_09985 ORFs (from the human SPM0211 and S23 strains, respectively) have less revealing hypothetical protein annotations. Likewise, annotations for the remaining non-core loci (P869_01660 and P869_04430 from the S23 strain) are also functionally uninformative, as both corresponding products have been designated as a hypothetical protein. Based on the annotations of these various accessory genes, nothing can be adequately deduced about their prospective roles in the gut-niche specialization of *L*. *ruminis*.

Of parenthetical interest, our finding that *L*. *ruminis* ATCC 27782 encodes for a maximum number of three SDPs seems to be in conflict with an earlier genomics study that had predicted the occurrence of ten such proteins in this bovine strain [21]. The loci in common with our study are those for the LrpA and LrpC pilus proteins (conceivably as well for LrpB) and LRC_RS00370, but no others were detected. Concerning the additional six loci identified in the other study [21], our examination of their predicted primary structures revealed that none have the C-terminal characteristics for a complete LPXTG domain region (S8 Table). Moreover, we noticed that for four of them (LRC_01690, LRC_16530, LRC_16780, and LRC_16790), they no longer exist in the NCBI database after the ATCC 27782-derived genome had been provided with new locus tags.

With the above in mind, it would seem that the potential variety of SDPs is not too extensive on the outer surface of the *L*. *ruminis* species. However, of the modest number of predicted SDPs, those that assemble into the LrpCBA pilus are likely to be advantageous and important for the adhesion of *L*. *ruminis* cells to the gut epithelium, and presumably for their inherent autochthonous character as well. Yet, one cannot exclude the possibility that among the other LPXTG-domained proteins whose function is not known, there still might be a useful adhesive role during host colonization. Further, given the host niche environment of the *L*. *ruminis* species, its nutritional sustenance weighs heavily on the ability to degrade and utilize various complex carbohydrates, particularly as found in the gut of herbivorous livestock [2, 56]. Thus, it comes as no surprise that two of the *L*. *ruminis* genomes (from bovine PEL65 and porcine DPC 6830) encode for a SDP whose putative function is a starch-acting pullulanase enzyme, though still somewhat puzzling that it is not more widespread among the genomes of the other animal-derived strains.

#### Flagellar and chemotaxis proteins

Often found on bacterial cell surfaces are long flagellar structures, and while these peripheral features primarily provide the driving force for cellular movement, they are also known to contribute to adhesion, biofilm assembly, and immuno-responsiveness (for detailed reviews, see [70, 71]). As mentioned beforehand, certain *L*. *ruminis* strains display a motility function (swimming, swarming, or tumbling) where one or more flagella are used. Presumably, the type of movement would be controlled as a response to external stimuli, which characteristically consists of numerous interactions between so-called chemotaxis proteins [70, 71]. As flagellation and chemotaxis are both complex processes that involve a large number of different proteins with a variety of functions, this would be reflected by the gene content of the *L*. *ruminis* pan-genome.

Among the pan-genome loci that are clearly annotated as having a flagellar-related function, there are an estimated 39 core genes, along with an additional nine accessory genes (S9 Table). From this it is evident that the genetics of *L*. *ruminis* flagellation is not open to much variation and that there is a highly conserved commonality in the genes needed for encoding the structural constituents and biosynthesis of a flagellum. Here, a majority of *L*. *ruminis* genomes (GRL1172, ATCC 25644, ATCC 27782, DPC 6832, PEL65, and S23) have only a single non-core flagellar gene, with one of the genomes (DPC 6830) having none at all. At a molecular level, this suggests that from strain-to-strain the flagella are structured and assembled quite similarly, and that any fundamental divergence from this in form or function is unlikely to be supported by the varying gene content within the accessory genome.

For the loci of the pan-genome that encode the chemotaxis proteins, there are a total of 16 core genes whose annotation predicts an involvement with flagellar chemotactic responses (S10 Table). The most conspicuous of these loci are those having functional annotations for regulating the frequency of tumbling, a phenomenon manifested when the rotation of the flagellum switches from an anti-clockwise to clockwise direction [70, 71]. Correspondingly, the *L*. *ruminis* core genome includes variously annotated loci for the cytoplasmic CheA, CheB, CheC, CheD, CheR, CheW, and CheY proteins. Found also prominent among the core genes are those encoding for a number of transmembrane methyl-accepting chemotaxis proteins (MCPs). These sensory receptor proteins are localized on the outer surface of bacterial cells and operate through the detection and binding of chemoattractants or chemorepellents in the surrounding environment [70, 71]. Ordinarily, this leads to a signaled activation of the Che proteins involving cyclical methylation and demethylation, and which ultimately controls tumbling behavior [70, 71]. It is thus clear that MCPs would be obligatory for the active functioning of the *L*. *ruminis* flagella. Interestingly, of the three to six non-core loci predicted to encode chemotaxis proteins most are having MCP annotations. Thus, for each of the various *L*. *ruminis* strains encompassing the pan-genome, there could be the functional potential for flagella to respond to differing stimuli that are representative of the intestinal milieu. Indeed, this might reflect a phenotype evolvability that tailors the adaptation of a *L*. *ruminis* strain to the specific environmental conditions in a particular host gut. Incidentally, it is tempting to speculate that the oddly resuscitated tumbling motility observed with the ATCC 25644 human strain *in vivo* [22] might rest with one or more anomalous or possibly defective chemotaxis proteins. This in itself can be seen as an alternative explanation to an earlier proposed model involving the transcriptive regulation of a flagellin gene [22].

Successful bacterial colonization in the host GI tract can be construed as the logical precondition of cellular persistence, and which can lead to an indigenous lifestyle. For the *L*. *ruminis* species, its potential for flagellar chemotactic movement is advantageous and can play a key adaptive role in occupying the gut autochthonously. For instance, through a response to certain attractants, flagella would likely be able to propel cells toward a favorable niche location containing abundant nutrients, reduced oxygen levels, and optimal attachment sites, such as what can be found throughout the folded crevices of the inner intestinal walls. Presumably, in reaching these deep and concealed locales, flagellum-directed motility will have helped prevent *L*. *ruminis* cells from becoming washed out along with the transiting luminal contents. Further, because chemotactic flagella are also able to sense and recognize repulsive stimuli, *L*. *ruminis* would be equipped to avoid the less ecologically hospitable aspects of the host intestine (e.g., oxygenated regions). By being flagellated on the outer cell surface, the various *L*. *ruminis* strains will have evolved an effective molecular strategy that supports an ecological adaptation to a very specific gut microenvironment, which can be viewed as also promoting a characteristic autochthonous presence within a given host.

### Fermentative and respiratory eco-fitness of anaerobic *L*. *ruminis*

In genomic terms, a strict gut anaerobe like the *L*. *ruminis* species has adapted itself remarkably well to the constraints of an oxygen-free ecological niche. However, owing to its intolerance of oxygen, *L*. *ruminis* should emulate the physiology of other obligately anaerobic bacteria by not producing the antioxidant enzymes that protect cells from reactive oxygen radicals [9]. Expectedly, none of the loci in the pan-genome of *L*. *ruminis* was found to be annotated as coding for superoxide dismutase and catalase, the enzymes responsible for degrading the superoxide radical and hydrogen peroxide, respectively (S1 Table). Thus, to be able to flourish under deoxygenated conditions, *L*. *ruminis* will have had to evolve the metabolic fitness to absorb energy substrates through either fermentative processes, anaerobic respiration, or possibly both.

#### Fermentation

**Fig 5:**
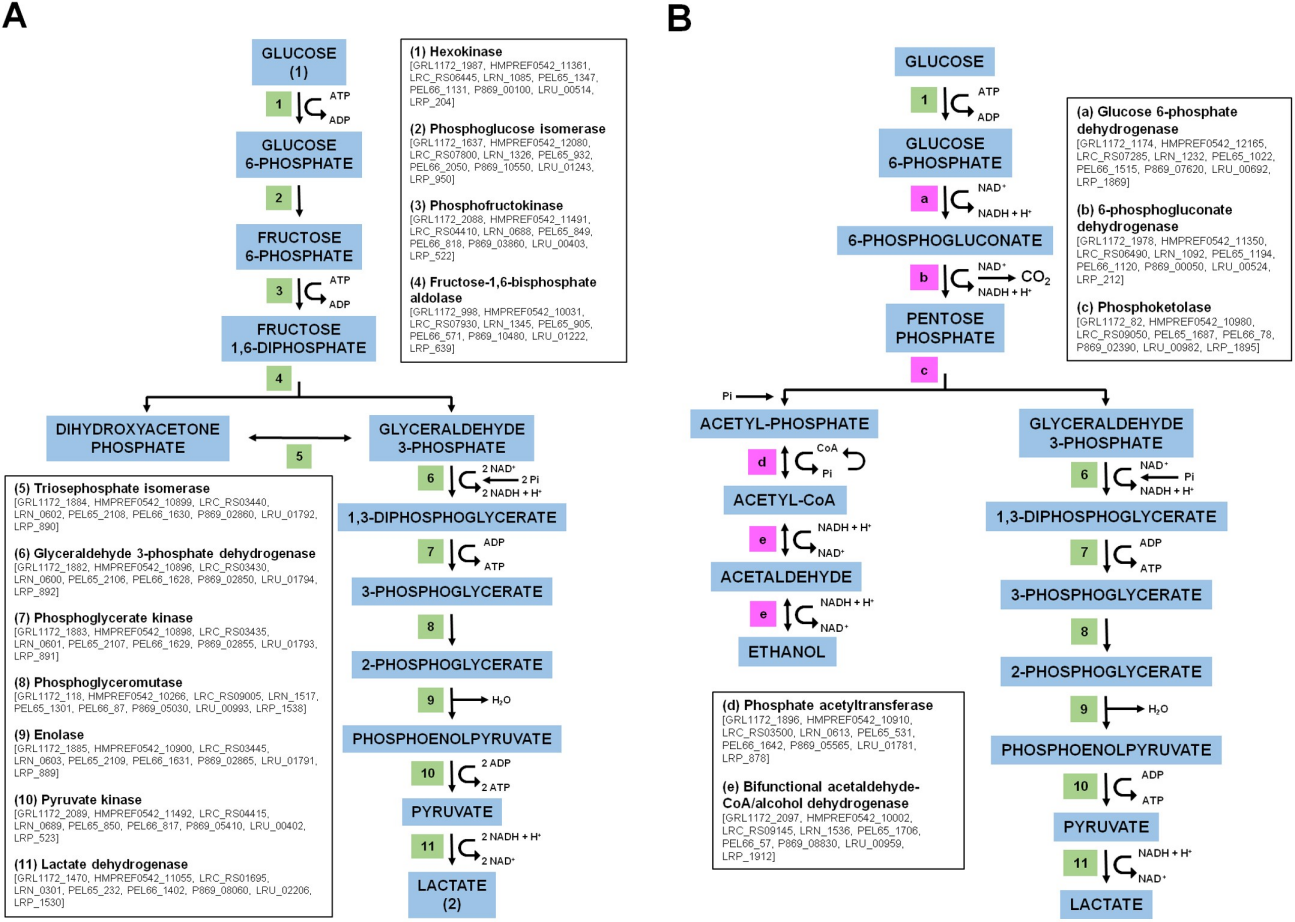
Schematic outline of the putative homolactic and heterolactic fermentative pathways in *L*. *ruminis*. Depicted is the glycolytic metabolism that can occur during homolactic fermentation via the Embden-Meyerhof-Parnas (EMP) pathway **(A)** or heterolactic fermentation via the phosphoketolase pathway (PK) **(B)**. Included are the various pathway reactions with their precursor metabolites and enzymes. Shown in the insets are the names of the glycolytic enzymes, either numbered (1–11) or alphabetized (a-e) along with the locus tags of their putative genes in the nine *L*. *ruminis* genomes. Enzymes that overlap in both pathways are numbered similarly.

Enough experimental evidence already exists to indicate that *L*. *ruminis* maintains an obligately homofermentative metabolism [17]. For this, cells will undergo lactic acid fermentation to generate cellular energy in a process that breaks down one molecule of glucose to yield two molecules of lactate [72]. Genomically, obligate homolactic bacteria are distinguished by the presence of fructose-1,6-bisphosphate (FBP) aldolase, a key glycolytic enzyme of the Embden-Meyerhof-Parnas (EMP) pathway through which hexose sugars are metabolized [72]. Our analysis of the *L*. *ruminis* pan-genome indicates that each of the nine strains has an ORF whose predicted product is annotated as a FBP aldolase (Fig 5A), which makes it part of the core genome (S1 Table). Further inspection of the pan-genome revealed that the annotated loci encoding all other EMP pathway enzymes, including lactate dehydrogenase for catalyzing the conversion of pyruvate to lactate, are as well found in the core-gene pool (Fig 5A). With such pathway-related genes in the core genome, this not only highlights an essential physiological role, but it also provides the genetic basis for the homolactic fermentation profile characteristically observed in *L*. *ruminis* cells.

Other *Lactobacillus* species are classified as either facultative or obligate heterofermenters and will instead metabolize glucose using the phosphoketolase (PK) pathway, but whereby lactate, ethanol, and CO_2_ are the end products [72–74]. In fact, the PK pathway is the same means that some lactobacilli use for fermenting pentose sugars [72]. One example is *Lactobacillus sakei*, which can grow on ribose as the sole source of carbon and energy [75]. In order for this to occur, ribose must be transported into the cytoplasm and then become phosphorylated. In *L*. *sakei*, these processes necessitate the involvement of an operon (*rbsUDK*) encoding the ribose transporter (RbsU), D-ribose pyranase (RbsD), and ribokinase (RbsK) proteins, and whose expression is regulated by the RbsR repressor protein [75]. In the case of *L*. *ruminis*, its own ability to catabolize ribose is the subject of conflicting studies, one reporting that it occurs in the bovine strain NRIC 1689 (also called ATCC 27780 or PEL65) [76], but another concluding that the same isolate along with several other strains shows no growth on this pentose sugar [18]. In this latter study [18], it was suggested that because the gene for a putative transaldolase (one of the enzymes in the pentose phosphate pathway) is not found in the genome of two strains (ATCC 27782 and ATCC 25644), this can be inferred as the possible reason why ribose is non-fermentable by *L*. *ruminis*. However, given that the foremost function of the pentose phosphate pathway is an anabolic one, a missing transaldolase enzyme might not necessarily represent an adequate explanation, particularly since pentose fermentation by heterolactic bacteria is mainly through the PK pathway [72].

To tackle this discrepancy, we examined our pan-genome data (S1 Table) to determine whether *L*. *ruminis* contains the genes that encode the necessary enzymes for a PK pathway. Loci for the full repertoire of PK pathway enzymes were detected in the *L*. *ruminis* core genome (Fig 5B), but with the exception of the all-important pentose-cleaving phosphoketolase, which was not found in the genome of the DPC 6832 strain. Of related interest, following the completion of this article, we became aware that the phosphoketolase gene is present in the *L*. *ruminis* DPC 6832 genome after its locus tags in the NCBI database were reassigned. Nonetheless, while it remains uncertain that these gene products are actively expressed and participate in a functional pathway that ferments pentose sugars, one might begin to speculate and reconsider whether *L*. *ruminis* is suitably placed in a group that contains obligately homofermentative lactobacilli. Yet, on the other hand, an examination of the *L*. *ruminis* pan-genome for the genes encoding the proteins involved with mediating active ribose transport had revealed that while annotated genes for the ribokinase (GRL1172_644, HMPREF0542_10159, LRC_RS07720, LRN_1311, PEL65_950, PEL66_2068, P869_06985, LRU_01270, and LRP_932) and ribose operon repressor (GRL1172_1281, HMPREF0542_12070, LRC_RS08440, LRN_1438, PEL65_1929, PEL66_741, P869_08795, LRU_01112, and LRP_465) proteins were identified within the core genome, those for the ribose transporter and D-ribose pyranase proteins were not present. Though other factors can be involved, one can justifiably argue the lack of growth on ribose by *L*. *ruminis* strains that others had observed [18] might be more attributed to the missing components of the ribose transport machinery. This in turn would serve to lessen any legitimacy that the bovine isolate of *L*. *ruminis* (NRIC 1689) is a ribose-fermenting strain [76]. Thus, if it is the situation that ribose (or xylose [18]) cannot be utilized by *L*. *ruminis*, it raises the question as to which type of pentose sugar would be transported into the cell and processed through a prospective PK pathway. However, were it otherwise to be shown that a particular *L*. *ruminis* strain can as well undergo a heterofermentative metabolism via the PK pathway using various pentose and hexose sugar substrates, this can be seen as an adaptive gain or benefit when competing in the gut microcosm, as it would offer a wider access to fermentable carbon and energy sources, particularly during periods of fluctuating nutrient availability. Presumably, the potential capacity for cellular growth using a more mixed fermentative metabolism would further help support the perpetuity of *L*. *ruminis* colonization within the host gut.

#### Anaerobic respiration

Contrary to their namesake, certain members of the LAB also have the genetic means of obtaining energy by respiration, whereupon lactate is no longer the main end product, but then provided that an exogenous source of both heme and menaquinone is available beforehand to cells [77]. In an anoxic environment, the absence of oxygen precludes its use as the terminal electron acceptor in the respiratory chain, and thus other oxyanionic compounds must be used instead, and for which a corresponding oxidoreductase enzyme is considered indispensable [78, 79]. Thus far, among the LAB only a few have the minimal number of necessary genes for an anaerobic respiratory metabolism, such as when either nitrate (*Lactobacillus plantarum*, *Lactobacillus fermentum*, and *Lactobacillus reuteri* [80]) or fumarate (*Enterococcus faecalis* [81]) is used as the final electron acceptor.

Generally, for bacteria that respire anaerobically using nitrate, this ability can be identified at the genomic level by the clustering of genes (*narGHI* operon) for a nitrate reductase complex, which itself consists of three subunits (NarG, NarH, and NarI) and is membrane-bound [78]. Still, for other bacteria, a single locus that encodes a monomeric soluble nitrate reductase can as well be found, and presumably, this form would represent the smallest structural and functional unit that mediates nitrate reduction [78]. Gene prediction within the *L*. *ruminis* pan-genome shows no evidence of what resembles the *narGHI* operon, but there is one ORF with a nitrate reductase annotation (LRC_RS08915 from ATCC 27782) (S1 Table). However, a closer inspection of its primary structure had revealed this ORF is likely mis-annotated. Based on the domain assignments from the Pfam database [69] (data not shown), the putative protein product of this ORF actually belongs to the amidinotransferase family, which as well includes the arginine deiminase enzyme. It should be noted that orthologous genes from other genomes in the pan-genome have these two types of annotations. Evidently, there is no genetic rationale in *L*. *ruminis* for carrying out anaerobic nitrate respiratory growth.

**Fig 6:**
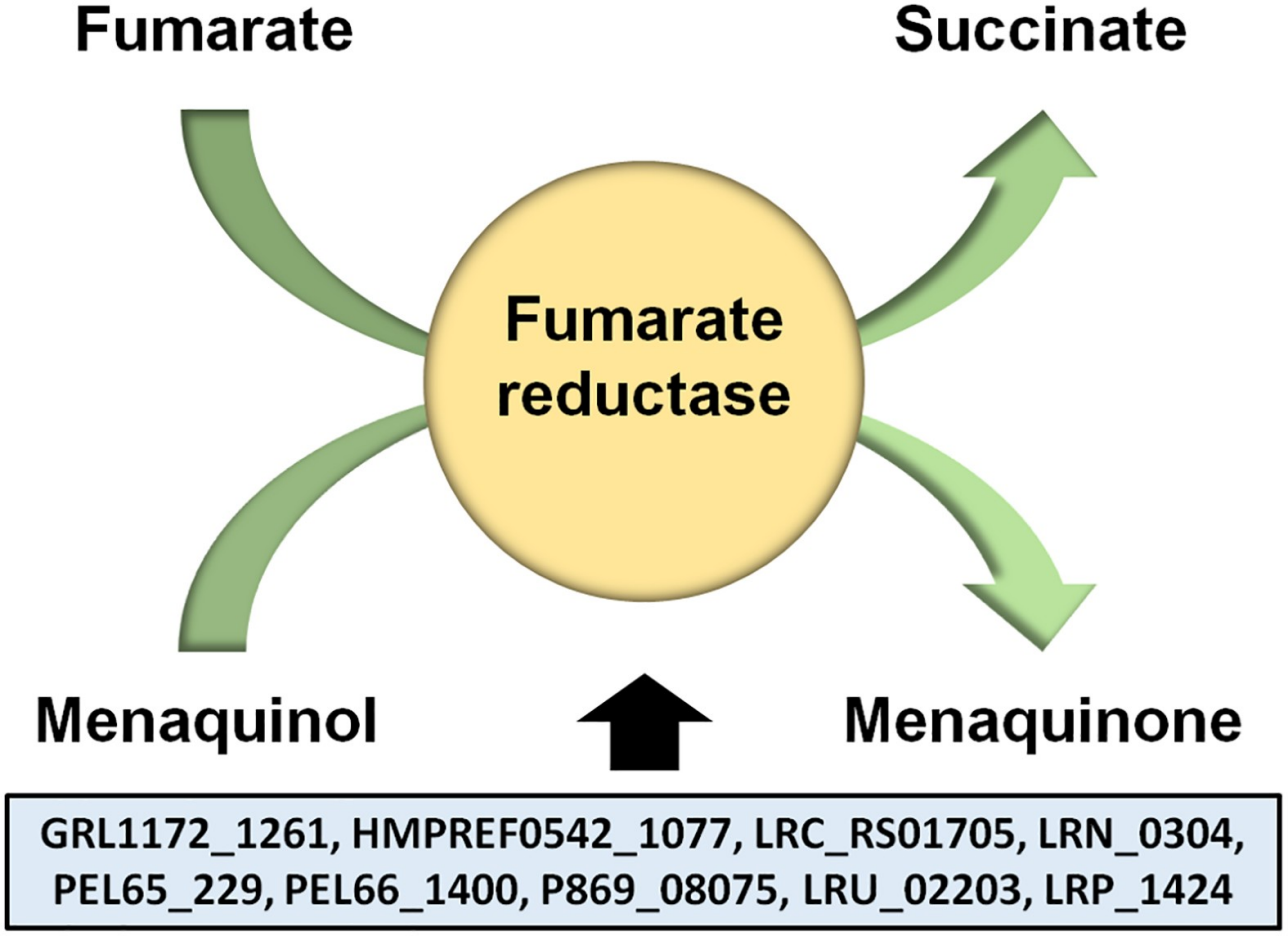
Schematic representation of the fumarate reductase-catalyzed reaction. Shown is the reaction catalyzed by fumarate reductase, where the reduction of fumarate to succinate is coupled with the oxidation of menaquinol to menaquinone. Included are the nine locus tags for the putative fumarate reductase gene found present in the *L*. *ruminis* core genome.

In contrast, our analysis of the pan-genome data did seem to indicate the possibility of fumarate respiration in *L*. *ruminis* (S1 Table). Here, we found ORFs in the core genome (GRL1172_1261, HMPREF0542_10777, LRC_RS01705, LRN_0304, PEL65_229, PEL66_1400, P869_08075, LRU_02203, and LRP_1424) whose annotation suggests that they encode for a fumarate reductase (Fig 6), the key enzyme allowing the use of fumarate as the terminal oxidant during anaerobic respiratory growth [79]. Following a BlastP search, we were more convinced of this identity, as its primary structure shows strong similarity with the flavocytochrome c fumarate reductases from other types of bacteria (data not shown). Since bacterial fumarate reductases are able to exist as both a membrane-bound or soluble enzyme, this is also differentiated in the way they are encoded in the genome. For instance, fumarate reductase in *Escherichia coli* is located on the inner surface of the cytoplasmic membrane and consists of four different subunits that form the catalytic (FrdA and FrdB) and membrane-embedded (FrdC and FrdD) domains, with their corresponding genes encoded within the *frdABCD* operon [79]. Alternatively, for the genus *Shewanella*, fumarate reductase is a soluble periplasmic enzyme, existing as a monomer and thus encoded by a single gene [79]. Likewise, in the LAB *E*. *faecalis*, it is recognized that only one gene encodes its fumarate reductase [81]. As for the putative fumarate reductase we uncovered in *L*. *ruminis*, its locus is not part of a gene cluster, thus suggesting that this enzyme is monomeric. Since the *L*. *ruminis* fumarate reductase gene was not found amongst our SignalP or TMHMM prediction data (S1 and S5 Tables, respectively), but was identified when using the SecretomeP program (S3 Table), it is reasonable to assume that this enzyme is not membrane-bound, and perhaps might even be non-secretable and confined to the cytoplasm. Nonetheless, even though anaerobic respiration is regarded as less energetically efficient than aerobic respiration, it still yields more energy than when bacterial cells use fermentative processes and, by comparison, will lead to increased cellular biomass and long-term survival [82]. In terms of metabolic versatility, any putative capacity of *L*. *ruminis* to respire anaerobically on fumarate instead of undergoing lactic acid fermentation would likely represent a competitive edge over the other non-respiring intestinal bacteria and a potential fitness benefit for autochthonous growth in the gut.

## Conclusions

In this present study, we used the pan-genomic approach to take an *in silico* appraisal of what might be the molecular basis for *L*. *ruminis* gut autochthony. Obviously, from the outset, there seems little doubt that the overall genetics behind the autochthonous growth of *L*. *ruminis* cells is multifaceted and involves many different physiological properties operating both independently and synergistically within the influencing context of the intestinal milieu, which itself includes a myriad of microbe-microbe and microbe-host interactions. Nonetheless, in an effort to puzzle out these underlying geno-phenotypes, we focused our attention on certain genes and proteins associated with the cellular surface morphology and fermentative/respiratory metabolism, which can be viewed collectively as ecologically relevant host colonization determinants in *L*. *ruminis*. For this, we have proposed plausible scenarios for how each of these molecular traits might have a functional role in making *L*. *ruminis* better adapted to the host intestine, such that a sustained colonization takes precedence over an impermanent one, and its “realized” niche is occupied instead of one that is directly associated with food, and thus transient. In all probability, there is a combination of proteinaceous surface structures, along with possible extra energy-generating pathway alternatives for an anaerobic metabolism, that help provide *L*. *ruminis* cells with a high fitness for the deoxygenated folds of the intestinal inner lining. Such genomic adaptations distinguish *L*. *ruminis* as a gut-persistent bacterium, but as well, an ecological specialist. However, even though the genetic content inferred from the pan-genome would indicate that *L*. *ruminis* is a rather resourceful and adaptable bacterium, it remains unclear why any number of strains has not yet been recovered from anoxic habitats other than the gut. This is suggestive that any inherent genomic plasticity is not meant for this species to spread out into new and different ecological territories. Rather instead, it might be more intended for the ability of the *L*. *ruminis* genome to quickly respond and adapt when changes occur in the dynamic intestinal environment and/or amidst the competitive microcosm of gut bacteria. Thus, despite being restricted to a particular niche locale, *L*. *ruminis* has evolved to its present status using a genetically resilient but flexible genome. Here, one can reasonably argue the point that by having an open and strong potential for acquiring different adaptive phenotypes, this will help *L*. *ruminis* cells perpetuate their autochthonous colonization of the GI tract.

## Supporting information

S1 Table Predicted gene content of the *L*. *ruminis* pan-genome. Compiled from the nine *L*. *ruminis* genomes is a list of locus tags and their functional annotations that define the gene content of the pan- (4301), core (1234), and accessory (3067) genomes. Locus tags of core (gray highlight) and strain-specific accessory (green highlight) genes are indicated.
(XLSX)

S2 Table SignalP-predicted proteins in the *L*. *ruminis* pan-genome. Protein sequences predicted from the loci in the *L*. *ruminis* pan-genome were analyzed by the SignalP 4.1 algorithm (http://www.cbs.dtu.dk/services/SignalP/) using the default settings for Gram-positive bacteria and a cut-off score of ≥ 0.45. Predicted products of ≤ 45 amino acids were excluded from the analysis. Locus tags for genes with SignalP hits are listed per *L*. *ruminis* genome. Core genes are highlighted in gray and accessory genes are without highlights. A numerical summary of core and accessory genes with SignalP hits is provided. (For one-to-one orthologous correspondence between the genomes, refer to the S1 Table.)
(XLSX)

S3 Table TatP-predicted proteins in the *L*. *ruminis* pan-genome. Sequences from the predicted products of the genes in the *L*. *ruminis* pan-genome were analyzed using the TatP 1.0 program at default settings (http://www.cbs.dtu.dk/services/TatP/). Predicted products of ≤ 45 amino acids were omitted from the analysis. Locus tags of core (highlighted in gray) and accessory (no highlights) genes with TatP hits are listed according to each *L*. *ruminis* genome. Total numbers of core and accessory genes with TatP hits are included. (For one-to-one orthologous correspondence between the genomes, refer to the S1 Table.)
(XLSX)

S4 Table SecretomeP-predicted proteins in the *L*. *ruminis* pan-genome. Predicted products of the loci in the *L*. *ruminis* pan-genome were screened for non-classical secreted proteins by using the SecretomeP 2.0 program set at default (http://www.cbs.dtu.dk/services/SecretomeP/). Predictions with the recommended SecP score of ≥ 0.5 identified possibly secretable proteins. Proteins of ≤ 45 amino acids were not part of the analysis. Listed are gene locus tags for each *L*. *ruminis* genome and their corresponding SecP score. The total number of SecP hits per genome is provided. (For one-to-one orthologous correspondence between the genomes, refer to the S1 Table.)
(XLSX)

S5 Table TMHMM-predicted proteins in the *L*. *ruminis* pan-genome. Putative gene-products from the *L*. *ruminis* pan-genome were examined for the presence of transmembrane helical regions by using the TMHMM 2.0 algorithm with default settings (http://www.cbs.dtu.dk/services/TMHMM/). Proteins of ≤ 45 amino acids were not included in the predictive analysis. Listed per *L*. *ruminis* genome are the gene locus tags and their corresponding number of predicted transmembrane helices (TMHs). The total number of TMHMM hits for each genome is given. (For one-to-one orthologous correspondence between the genomes, refer to the S1 Table.)
(XLSX)

S6 Table PRED-LIPO-predicted proteins in the *L*. *ruminis* pan-genome. Predicted gene-products from the *L*. *ruminis* pan-genome were analyzed by the PRED-LIPO program (default settings) (http://biophysics.biol.uoa.gr/PRED-LIPO/) to identify putative lipoproteins. Proteins of ≤ 45 amino acids were not used in the predictive analysis. Locus tags of core and accessory genes (gray and no highlights, respectively) with PRED-LIPO hits are listed for each *L*. *ruminis* genome. The total number of PRED-LIPO hits as per core and accessory genome is shown. (For one-to-one orthologous correspondence between the genomes, refer to the S1 Table.)
(XLSX)

S7 Table CW-PRED-predicted proteins in the *L*. *ruminis* pan-genome. For predicting possible sortase-dependent proteins (SDPs), the *L*. *ruminis* pan-genome loci were analyzed by the CW-PRED program (default settings) (http://bioinformatics.biol.uoa.gr/CW-PRED/), which detects the presence of an intact C-terminal LPXTG cell wall-anchoring domain. Proteins of ≤ 45 amino acids were left out of the analysis. Locus tags of genes with CW-PRED hits and their corresponding annotations are listed for each *L*. *ruminis* genome. CW-PRED hits for core-gene locus tags are highlighted in gray. Amino acid sequence for the predicted C-terminal LPXTG domain regions (beginning with the LPXTG motif in red font) is included. (For one-to-one orthologous correspondence between the genomes, refer to the S1 Table.)
(XLSX)

S8 Table Predicted sortase-dependent proteins in the genome of *L*. *ruminis* ATCC 27782. Listed are the gene locus tags of predicted sortase-dependent proteins (SDPs) in the genome of the bovine-derived *L*. *ruminis* ATCC 27782 strain, as reported by Forde and colleagues (see [21]) (*), and as presently available in the NCBI database (**) and used in the present study. Locus tags for SDP loci that were part of the *L*. *ruminis* core genome are highlighted in gray. Provided is the amino acid sequence for all putative SDPs 21, with the LPXTG-like motifs indicated by red font.
(XLSX)

S9 Table Putative flagellum-related proteins in the *L*. *ruminis* pan-genome. Locus tags of flagellum-related genes along with their respective annotations are identified manually and listed for each *L*. *ruminis* genome. Locus tags for genes belonging to the core genome are highlighted in gray. Total numbers of flagellum-related core and accessory genes are included.
(XLSX)

S10 Table Putative chemotaxis-related proteins in the *L*. *ruminis* pan-genome. Locus tags of chemotaxis-related genes and their corresponding annotations are found by hand and listed for each *L*. *ruminis* genome. Gray highlighting indicates those gene locus tags in the core genome. The total number of chemotaxis-related core and accessory genes is provided.
(XLSX)
